# Trust, Communication, and Community Networks: How the CORE Group Polio Project Community Volunteers Led the Fight against Polio in Ethiopia’s Most At-Risk Areas

**DOI:** 10.4269/ajtmh.19-0038

**Published:** 2019-10

**Authors:** Katherine V. Stamidis, Lydia Bologna, Filimona Bisrat, Tenager Tadesse, Fasil Tessema, Elizabeth Kang

**Affiliations:** 1 CORE Group Polio Project, Washington, District of Columbia;; 2 CORE Group Polio Project/Ethiopia, Addis Ababa, Ethiopia;; 3 Department of Epidemiology, Jimma University, Jimma, Ethiopia;; 4 Johns Hopkins Bloomberg School of Public Health, Baltimore, Maryland

## Abstract

The last case of wild poliovirus in Ethiopia was reported in 2014. Until the disease is eradicated globally, the risk of reimportation remains high. In 1999, the CORE Group Polio Project (CGPP) began its community-centered polio eradication efforts in Ethiopia, using community volunteers (CVs) to ensure that no child has missed polio vaccine. This article documents the efforts of CVs and highlights innovative strategies, successes, and contributions. Qualitative data were collected from the CGPP implementation areas in 85 border *woredas* (districts) of Benishangul-Gumuz; Gambella; Oromia; Southern Nations, Nationalities, and Peoples’ Region; and Somali. A total of 151 in-depth interviews were conducted with CVs, parents, CGPP partners, and project stakeholders. Results of the study showed that CVs secured the buy-in of community members through open and fair eligibility and selection processes, thereby ensuring representation of community needs and perspectives. Community-driven participation consisted of identifying and choosing credible, trusted individuals who were willing to actively engage as caretakers of the community. Community volunteers then received specialized training and supportive supervision to build and expand their command of child health and vaccination information and interpersonal skills, fortifying the legitimacy of health messages and supporting the community’s sense of collective efficacy. The robust network of CVs built by the CGPP continues to effectively reach the most remote, rugged, and underserved areas of Ethiopia. Stakeholders credit the CGPP with playing a significant role in keeping Ethiopia polio-free and increasing the population coverage of polio and routine immunizations.

## INTRODUCTION

Poliomyelitis (polio) is a highly infectious viral disease that can invade the central nervous system, causing irreversible paralysis. There is no cure for polio, but it is preventable through immunization. In low- and middle-income countries, immunity is gained primarily through several rounds of repeated vaccination with routine oral polio vaccine (OPV) and at least one full dose of inactivated polio vaccine, which is administered by intramuscular injection, or with a fractional (one-fifth) dose given intradermally. In 1988, the World Health Assembly resolved to work toward the eradication of polio. Just 8 years earlier, the World Health Assembly had declared smallpox eradicated—the only infectious disease holding this distinction so far. To eradicate polio through surveillance and vaccination, the Global Polio Eradication Initiative (GPEI) began its work 30 years ago to tackle a disease that was paralyzing 1,000 children every day across the globe. Today, the total number of polio cases has decreased by more than 99% worldwide.^1^ Yet, despite these promising results, there are remaining strongholds of at-risk populations in remote, insecure, and hard-to-reach areas in the three endemic countries of Afghanistan, Pakistan, and Nigeria.

After the signing in Cameroon of the Yaounde Declaration on Polio Eradication in 1996, Ethiopia joined the GPEI and immediately adopted its strategic recommendations.^2^ In the same year, the Ethiopian Federal Ministry of Health (EFMOH) conducted its first Subnational Immunization Day (SNID) by targeting 2.5 million children younger than 5 years (hereafter under-five children) in nine major cities in an effort to significantly increase the number of children vaccinated with four doses of OPV, the benchmark for full coverage. Before the SNID campaign, the percentage of under-five children who had been fully vaccinated against polio was only 35%.^3^

The last indigenous case of wild poliovirus (WPV) in Ethiopia was reported in January 2001.^4^ From February 2001 to December 2004, Ethiopia remained polio-free. However, between December 2004 and October 2006, there were four importations of WPV from Somalia and Sudan to Tigray, Amhara, Oromia, and Somali states. Children in these regions were highly susceptible to polio because of suboptimal routine immunization coverage levels in these remote areas.^5,6^ Children living in major cities were much more likely to have enough protection from OPV. However, children living in rural areas and pastoralist populations were and still are vulnerable. Currently, only 20% of Ethiopia’s population live in urban areas.^7^ The last recorded case of reimportation of WPV was in January 2014.^8^

In 1999, the CORE Group Polio Project (CGPP) began its community-based program to prevent, detect, and respond to polio transmission. As is the case in all CGPP countries, Ethiopia’s community volunteers (CVs) have been the crucial link to strengthening disease surveillance and knowledge, increasing vaccine coverage, and providing basic health information to improve health outcomes in hard-to-reach areas or in populations that are resistant to participation in polio immunization campaigns. The CGPP’s Ethiopian CV program is discussed elsewhere in this series.^9^

Rugged terrain, widely scattered population settlements, weak health service systems, limited participation of key community members in planning of vaccination activities, and well-traveled porous borders with polio-endemic areas pose the greatest challenges to control polio transmission in Ethiopia. These barriers result in low community awareness about polio vaccination and acute flaccid paralysis (AFP) surveillance.^10^ Evidence has demonstrated that approaches using CVs help resolve misconceptions surrounding vaccines and increase the accessibility of remote communities to routine vaccinations through referrals and mapping of communites.^11^ Ethiopia, according to the GPEI, continues to be at risk for poliovirus importation or re-emergence due to low immunity and poor disease surveillance. To minimize these risks, CGPP Ethiopia currently positions more than 12,000 CVs along and across the borders between Ethiopia and South Sudan, Kenya, and Somalia to support immunization services through health education, defaulter tracing, and identification of children needing vaccination. Community volunteers also comprise a strong network for disease surveillance.

Gains in immunization coverage are attainable by building the community’s trust through meaningful contact as part of a larger strategic effort. In India, for example, a successful social mobilization program called SMNet used community mobilization coordinators to increase rates of full immunization for children.^12,13^ In Somalia and Ethiopia, house-to-house visits by community health workers (CHWs) and mobilizers have helped improve vaccination coverage rates, especially for nomadic and pastoralist† populations.^14–17^ Community participation has also increased immunization coverage in urban slum areas.^18^

Ethiopia’s Health Extension Program began in 2003 to address the shortage of health-care providers in rural areas and improve health outcomes to meet the Millennium Development Goals. A defining aspect of the Health Extension Program was the establishment of the health extension worker (HEW) cadre. HEWs receive 1 year of training in health education and communication, hygiene, and environmental sanitation, disease prevention and control, and family health.^19^ The EFMOH committed to implementing the Health Extension Program nationally, with a planned integration of HEWs within the health system to improve quality and promote retention.^19^ Health extension workers are government employees who are provided with a regular salary and benefits by the government.

The CGPP CVs were established in 2003 to supplement the work of the government-sponsored HEWs, by providing education on vaccine-preventable diseases and mobilizing people to use immunization services. Community volunteers also actively search for and report cases of the three types of diseases under surveillance: AFP, measles, and tetanus. In addition, they identify newly pregnant women, newborns, and those in need of immunizations and track them accordingly.

In 2012, the government of Ethiopia introduced female Health Development Army (HDA) volunteers for social mobilization and health education with the intention of replacing all the other existing volunteer community health workers (but not the salaried health extension workers). In pastoralist areas, however, HDA volunteers were unsuccessful. The CGPP responded to this pressing need by providing CVs in many of these pastoralist communities. Each kebele (subdistrict) in the CGPP implementation areas has between three and five CVs selected and trained to support the HEWs. The government HEWs and the CGPP CVs combine efforts in an attempt to improve health outcomes for pastoralist communities.

The role of CVs and HDA volunteers is the same: to bridge the gap between the lower level health-care system (health posts and health centers) and the community. Both CVs in CGPP project areas and HDA volunteers who work elsewhere educate and mobilize the community for vaccination, health service utilization, and they support HEWs during routine immunization and immunization campaigns. The synergy between CVs and HDA volunteers produces greater coordination and reach of immunization and surveillance activities, and it also allows for a more robust and supervised community mobilization and surveillance system. Community volunteers and HDA volunteers meet monthly with the HEWs to plan, report, and receive updates on immunization and surveillance-related information. Working alone, HEWs would be unable to provide coverage to hard-to-reach areas without the support of these CVs. Health extension workers are responsible for a population of 2,500–5,000 people, many of whom may live several hours away from the health post where the HEW is based.^10,20^

The CORE Group Secretariat and its implementing partners are supporting the government health system in 85 hard-to-reach districts in five regions of the country. The CGPP supports immunization and surveillance activities—deploying CVs as well as training HEWs and other health workers on various topics including community-based surveillance, cold chain management, interpersonal communication, and data management. The CGPP also provides fuel for outreach activities, kerosene for refrigerators used to store vaccine, maintenance of motorcycles for routine immunization activities, vehicles to support immunization outreach, supplies, and per diems (daily financial incentives) to CVs, mobilizers, vaccinators, and guides during polio campaigns. The secretariat offers technical support and on-the-job training for immunization and surveillance, and it provides supportive supervision visits at the regional, zonal, and district levels.

The most recent CGPP evaluation survey data, collected in 2017 in CGPP implementation areas of Ethiopia, show demonstrable progress facilitated by CVs between 2012 and 2017. Oral polio vaccine 3 coverage increased from 40.6% to 73.2%, and the percentage of fully immunized children nearly doubled from 24.7% to 43.6% (fully immunized calculated according to the immunization cards kept by caregivers).^21,22^ Relatedly, 58.9% of caregivers reported that they had received information about the most recent polio campaign from CVs; this is an increase from a baseline of 41.3% in 2012. In addition, a higher percentage of caregivers received information about polio from CVs in 2017 (58.7%) than in 2012 (31.8%).^21^ Community volunteers have made a significant contribution to the elimination of polio in Ethiopia. This article examines the evolution of CVs in Ethiopia and the challenges they overcame to develop and implement a successful program.

## METHODOLOGY

### Study design.

The data for this article were originally collected for the CGPP Endline Evaluation of programmatic activities between 2012 and 2017. Qualitative data were collected through in-depth interviews. This method of data collection was chosen because it allowed participants’ freedom in answering and yielded fuller in-depth perspectives on a wide variety of topics.^23^ In Ethiopia, interviews were conducted during July and August 2017.

### Study setting.

Data were collected from CGPP implementation areas in the 85 border *woredas* of Benishangul-Gumuz; Gambella; Oromia; Southern Nations, Nationalities, and Peoples’ Region; and Somali regions. These pastoralist areas were selected because of their ongoing inclusion in the CGPP programming, with the data collection falling within the established program evaluation framework.

### Recruitment and data collection.

A local research team, comprised of university staff and postgraduate students who had earned a minimum of a master’s degree, received training in data collection methods, interviewing techniques, and ethical considerations. A local consultant and 10 supervisors/facilitators monitored data collection activities. The team conducted 151 interviews across 15 zones in the five regions of Ethiopia where the CGPP is active.

Interviews were held with CVs, parents, CGPP partner organizations, and stakeholders. Purposive sampling was used to recruit participants. Community volunteers and HEWs helped to recruit caregivers who had pertinent information to share. Mothers (30 participants) and fathers (30 participants) who had at least one under-five child were selected for interviews. Community volunteers (30 participants) who had been with the CGPP for at least 1 year were selected for interviews in each zone. Two CGPP partner staff and key stakeholders (*woreda* and zonal health officers) were selected from each zone. One high-level stakeholder was selected from each of the following organizations that were also active in the CGPP implementation areas: the Ethiopian Ministry of Health (MOH), United Nations Children’s Fund (UNICEF), the WHO, and the Rotary International. The geographic distribution and types of interviews are shown in Table 1.

**Table 1:** Types and locations of interviews conducted

Type of participant	Geographic area					Total
	Somali	Gambella	Benishangul	Oromia	SNNPR*	
Community volunteers	10	6	6	4	4	30
Mothers	10	6	6	4	4	30
Fathers	10	6	6	4	4	30
CORE Group Polio Project partners	10	6	6	4	4	30
Low- and high-level stakeholders†	8	5	6	4	4	31 (4 high-level, and 27 low-level)

* Southern Nations, Nationalities, and Peoples’ Region.

† All high-level stakeholder interviews were conducted in Addis Ababa.

Researchers developed semi-structured interview guides for each group of participants; the guides contained overlapping questions to triangulate responses on the same topics and questions. The research team interviewed all consenting participants in a quiet, private location for about 45–60 minutes, allowing ample time to explore topics and give detailed answers. The interviews were conducted in the local language and recorded. The interviews explored several key themes: the impact of CGPP CVs on changing the tide of polio transmission, building trust, and the qualities that made CVs successful.

### Data analysis.

All transcriptions followed the same protocol: use of the local language for the interviews, removal of identifying information, and final translation into English. Analysts used ATLAS.ti qualitative software 6.2 (ATLAS.ti Scientific Software Development GmbH, Berlin, Germany) to organize and code the data.

The purpose of the final analysis was to understand the impact and contributions made by CVs in the CGPP implementation areas in Ethiopia. A content analysis identified central themes, categories, and subcategories. A list of pre-prescribed codes was developed based on the interview guides used. Researchers reviewed transcripts multiple times to identify recurring themes, ideas, and patterns.

### Ethical considerations.

The qualitative data used in this analysis were collected alongside survey data for monitoring and evaluation purposes as part of the 2017 CGPP Endline Evaluation, rather than for research purposes. The CGPP Secretariat in Addis Ababa provided each of the zonal and regional health offices with an official letter stating the purpose of the evaluation surveys and interviews. The purpose of the interviews, the voluntary nature of responses and interviews, and the ability to refuse or stop an interview were explained to the participant. Voluntary informed consent was verbally obtained with each participant before interviews. No names are reported or linked to responses, and identifying information and responses were kept confidential.

## RESULTS

### Who are CVs and what are their responsibilities?

The CVs are selected from the communities that they serve in and have a keen understanding and knowledge of the culture, values, and norms in the community. They are selected with participation of the *woreda* health offices, partner organizations, and most importantly, the community. To be effective, it is important for these CVs to be accepted and respected by the community. Community volunteers must be able to model positive behaviors and communicate in the local languages. Literacy is not a requirement, particularly in very remote and rural areas of Ethiopia. Most CVs are women (50–75%) depending on the locale. Community volunteers receive small incentives for their roles, including umbrellas, bags to carry materials, and an apron with the organization’s logo. For travel and *woreda-*level review meetings, they receive per diems. During active case searches and reporting, CVs are provided with mobile phones and solar lights to enable them to record and report cases.

After their selection, CVs typically participate in a 3-day training before beginning their participation. This training focuses on surveillance for AFP, measles, and neonatal tetanus, support for supplemental polio and routine immunization activities, and registration, tracking, and referral of pregnant women and newborns to health facilities for medical care. The CGPP partners provide refresher trainings and knowledge building through supportive supervision visits to CVs and through monthly meetings with HEWs. These supervision visits are critical because they allow the activities of CVs to be monitored, and they also give CVs a forum to ask questions and obtain needed support and information. Community volunteers go house-to-house in their communities, providing health education, tracking pregnant women and newborns, making referrals, tracking immunization defaulters and referring them to health facilities, and searching for cases of AFP, measles, or tetanus. During supplemental immunization campaigns against polio, they mobilize families to increase the demand for immunization.

### Building trust and empowering communities.

Participants explained that at the inception of the CGPP, the use of CVs to share knowledge about polio vaccination was met with skepticism and disdain from communities. Families struggling in low-resource settings were focused on survival and did not view polio immunization as a priority. The work of CVs was made difficult not only by the low priority given to polio immunization but also by misinformation, the spread of rampant misconceptions about vaccines, and harmful cultural practices.

“Initially, the community insulted me when I started to gather mothers and visit house to house. Mothers said, ‘You repeatedly come to our home. So, do you want to be a doctor?’ I was depressed and consulted the HEWs (who) advised me this is a common phenomenon while you work in the community. I convinced myself to continue with these attempts to change their minds on vaccination. After a long time, I have now finally received recognition in the community.” (CV, Aguna)

Initially, CVs encountered tension, disrespect, and lack of trust. Some CVs faced inhospitality; other CVs considered quitting their roles. Yet, because of a belief in importance of their work, they persisted. Over time, they slowly established trust, built rapport, and improved relationships with the families in their communities.

Today, the relationships of CVs with their communities and families are symbiotic, close, and congenial, according to reports from multiple sources. Both CVs and community members reported newly found empowerment as a result of greater knowledge about vaccination. Community volunteers use their own behavior to model positive action for their communities, and they were able to convince other parents to do the same, creating demand for vaccination.

“We did not believe the community workers about vaccination unless we see them do so. So, since they did show us by having their own children vaccinated, we came to trust our community workers.” (Mother, 22, Metekel)

Equipped with training in interpersonal communication and social mobilization skills, CVs used their accumulated knowledge on vaccines and child health to win over skeptical neighbors, caregivers, and parents. Over time, CVs were able to establish long-standing relationships with families, making interactions more amiable and fruitful. Moreover, emboldened CVs used persuasion and negotiation to change behaviors, leading to great satisfaction, recognition, and empowerment for the CVs.

“I am very happy for being part of such programs because there were a lot of problems among mothers during their pregnancy and childbirth. Now we (CVs) and our families benefit from the information we receive first and then our community also benefits as a result of the information we provide. This gives me satisfaction.” (CV, Assosa)

One father recounted the important contributions of CVs, tying their work to the noticeable decline in the incidence of childhood disease.

“Community awareness is better. This is due to the fact that health workers are very much engaged in house-to-house visits to provide education on immunization. The other reason is that the community is observing the benefit of vaccination.” (Father, Kellem Wollega)

Parents grew increasingly receptive to messages from CVs on vaccination and child health because they began to see the number of cases of polio, measles, and tetanus decline in their communities. Eventually, CVs, once approved in their communities, were embraced and applauded as champions of health and elevated to places of respect within their communities.

### Trusted sources of information and awareness about vaccines and child health.

Community volunteers and HEWs were consistently identified by parents as the most trusted sources of information about vaccines and child health. For mothers, CVs and HEWs represented the primary sources of information on vaccination and child health within their communities.

“Those community mobilizers are trusted. The information delivered by community mobilizer are accepted and trusted in the community. When they go to the community, they introduce information about vaccination, and people listen.” (Woreda EPI coordinator, Nuer)

Women discussed the credibility and trust they had in CVs and the ability of CVs to provide information through regular interactions and at special events such as coffee ceremonies.

“We trust the information about vaccines provided by Community Volunteers, HEWs or community leaders. When they share this, we get ready to go to the vaccination site. If we get information from our coffee ceremony, we take advantage and our children get vaccinated.” (Mother, Metekel)

Community volunteers meet the mothers at their homes and organize “mothers’ groups,” a safe space for women to learn about the importance of vaccines and other positive health-seeking behaviors. Some mothers commented on the freedom and opportunity these groups gave them, and their power in changing potentially harmful traditional norms. Other women, particularly in remote areas, expressed the need for more support and information, and asked for more regular visits with CVs and other health workers. Although some women found mothers’ groups to be helpful, others found that their other household burdens made it difficult or impossible for them to participate.

“I did not participate because of my own personal problem. I do not have an older child who cares for my kids. I have to fetch water. I have to collect firewood. So, I do not have time to participate in such meeting, but I hear about it from others who participated.” (Kamashi, Mother)

Fathers cited CVs and HEWs as trusted sources, but relied more on health posts, religious leaders, and doctors or nurses for vaccination information. Although not the sole source of information, fathers held CVs in high regard and viewed them as valuable assets.

“Information on vaccination is received during campaigns from the health extension workers, sometimes from schools, churches and spiritual places, or health posts with nurses. When people hear such information, they are willing to vaccinate their children, as opposed to previous years. They participate to vaccinate their children.” (Father, Kelem Wollega)

Mothers and fathers described CVs as having a much broader reach beyond polio immunization. Community volunteers leveraged these relationships and interactions with communities and families to share other health knowledge. They provided information and referrals for people living in places where health facilities and health professionals were not readily available.

### Changing community attitudes, practices, and health.

Long-serving CVs recounted changes in attitudes about vaccination. Intensive engagement and persuasion eventually replaced parental resistance with acceptance. Providing accurate knowledge on the benefits of polio and other routine immunizations, understanding social norms of the community, and investing time to listen and respond to parents’ concerns contributed to shifting attitudes and practices and ultimately to changing behaviors. Both parents and CVs spoke of noticeable improvements not only in vaccination coverage but also in the overall health and well-being of children and families in their communities.

“Since this program started, there are good and positive changes in child health. Most of the childhood illnesses are declining. There are a lot of changes. I have seen the changes in my own family and the community too … There are also changes in attitudes.” (CV, Assosa)

Increased knowledge among both mothers and fathers contributed to the demand for vaccine among communities. The push to convince parents to vaccinate became unnecessary as parents began making independent decisions to seek out vaccination opportunities.

“Parents are vaccinating their children on time. The community informs me if any vaccine-preventable diseases occur in our locality. All mothers know about vaccination benefits and risks of not vaccinating their child. The awareness about vaccination has increased. Mothers take their children to health facilities for vaccination by themselves. Demand has also been created in the community about antenatal care. Awareness has also been created even among men. Men are now taking their children to the health post and to campaign posts for vaccination.” (CV, Olegn)

Community volunteers highlighted improvements in timely vaccination and attributed this to increased knowledge about the benefits of vaccines in preventing disease.

“The CGPP communities have changed and have become healthy and productive. Formerly, the area was well known for its malaria, tuberculosis and measles, but now these diseases have ceased. For instance, for a long period of time, there have not been any children with polio symptoms in our communities, though there have been rumors of such cases in other areas. There has been a great change in attitudes about vaccination in the community. Since the area is sparsely populated and it has been very difficult to offer the services, we are now thankful to the [CGPP] project because these challenges are solved through its innovative approaches and mobilization of resources. Therefore, these are the changes that have been produced over time by the comprehensive efforts of the [CGPP] in cooperation with EECMY [an implementing partner].” (CV, Majang)

Community volunteers see their role as an information bridge between health posts and the community members they serve. For community members, particularly those in isolated rural areas, CVs are the first and only source of information, placing them in a unique position to improve health outcomes.

“The strength of CVs is their capacity to link local communities with their respective health posts [which are often several hours away]. The CVs are part and parcel of the local community and they can easily understand the outlook of the local community. This is the biggest strength of CVs.” (CV, Chereti)

The CGPP provides training to CVs and others in the health systems of Ethiopia. Although all CVs interviewed reported receiving some training, the amount, quality, and adequacy of that training varied significantly from one CV to another. The request for additional training was a universal response during interviews. Some CVs requested refresher training on polio, routine immunization, and surveillance, whereas others requested training and support in other areas beyond immunization and surveillance.

“Yes, I need to have additional training on mothers’ health care, sexually transmittable diseases, and on skills of first aid and delivery since this helps me to give advice to mothers and fathers about how they can protect themselves and how they can help each other during emergencies and during deliveries. I also need training on first aid skills in case something happens to help the community until a health worker arrives. Lastly, but most importantly, I also need refresher training on those topics I have been trained on previously.” (CV, Majang)

### Reaching the unreachable through community networking, collaboration, and surveillance.

In highlighting the strengths and accomplishments of CGPP’s work in Ethiopia, key stakeholders and partners universally pointed to the CGPP’s ability to reach the “unreachable” with vaccination education, community outreach, surveillance, and vaccinations. The CGPP does access hard-to-reach places where most stakeholders, partners, and the government have been unable to ignite change.

“The CGPP is delivering information and vaccinations [with paired vaccinators] in hard-to-reach areas or in the place where even the government is not providing any service.” (Woreda health office vaccination coordinator, Majang)

“The CGPP has an established structure which stretches down to the community level. Such things have made a great contribution to the system in those regions. In addition, their project has been implemented in hard-to-reach, high-risk areas and in emerging regions, such as Afar, Somali, and Benishangul. Moreover, resources were mobilized to these areas with due attention to eradicate polio.” (UNICEF, immunization specialist, Addis Ababa)

The CGPP has established an extensive network of strong community partners with a presence at all levels, enabling the CGPP to fulfill its mission and, by extension, to provide crucial support to all stakeholders and partners.

“As UNICEF, we do not go beyond zonal levels; we work at regional and zonal levels - not beyond that. However, the CGPP works at the community level. And when you map the resources and organizations working at the community level, you always find the CORE Group [the CGPP].” (Immunization specialist, UNICEF, Addis Ababa)

This network has created access to communities that were once inaccessible to polio immunization, providing a platform to respond to other health challenges. This network can be harnessed in the future to strengthen health systems ad referrals.

“Personally, I believe they do have a strong platform and they do have the capacity to reach to the lower level in hard-to-reach areas. As much as possible, we want to utilize their platform, and we have been utilizing it.” (MOH, EPI team leader, Addis Ababa, Ethiopia)

Stakeholders credited the CGPP’s unique ability to create a system that works in conjunction with the government system. This has resulted in strengthening health systems by using limited resources effectively and efficiently for greater reach and heightened impact.

“The program [CGPP] is working in hard-to-reach areas - that is a big contribution. By working and establishing a system in the areas where the government health system has been weak … they are working along the [national] borders. Establishing and strengthening the system in such areas has been a big contribution.” (Immunization specialist, UNICEF, Addis Ababa)

The CGPP has used an approach that is strongly rooted in the communities where it works. Community buy-in, involvement, and trust have ensured the success of the program.

“First of all, creating good relationships was important. There is a proverb called ‘kefitfitu fitu’ that explains, to create sustainable partnerships we should first create good relationships. [In this program], creating a good relationship was important first before deciding how to work together efficiently.” (Health program officer, Borena)

In addition, community, stakeholder, and partner engagement at multiple levels has been a crucial component of the CGPP’s success in Ethiopia. The partnerships and networks formed will be key in the transition of polio resources and assets once global eradication has been achieved.

Slowly, through strengthening partnerships and through the use of intensive community mobilization, communities increased their knowledge of vaccines. Vaccine uptake increased, and immunization coverage began to climb, as evidenced by interviews and CGPP evaluation data. These achievements were possible because the CGPP put resources into training and capacity building of local health facility staff on routine immunization, timeliness of vaccines, and supporting cold chain strengthening to ensure that the demand for vaccines is met with an adequate supply of vaccine and staff.

The CGPP has been able to harness the power of communities to change the local narrative around vaccination and to provide support for the improvement of other health-seeking behaviors.

“On polio eradication, our main contributing partners are WHO, UNICEF, and the CORE Group Polio Project. All have contributed to raising the immunization coverage. But the CGPP has played a significant role for polio eradication in hard-to-reach areas. First, they work at lower levels outside of health facilities and have many local implementing partners that can take into account the local context. They also providing support for routine immunization.” (MOH, EPI team leader, Addis Ababa, Ethiopia)

### How did the CGPP CVs become successful?

Community volunteers and CGPP partners spoke about the qualities that have made CVs successful in their communities. They cited training, knowledge about polio and health, supervision, building trust in communities, consistent one-on-one household contact, and understanding of the community as key ingredients of success. Training and learning opportunities, in particular, have prepared CVs to be advocates for polio and routine immunization and to proudly serve as champions of child health.

“What makes us successful is the trainings given to us and to other Community Volunteers and the close relationships that the Community Volunteers have with their communities.” (CV, Majang)

Developing knowledge in areas outside of polio has elevated the roles of CVs and made them more trusted sources of information. Mothers depend on CVs for information regarding other child health issues, such as advice on breastfeeding and timely vaccination, defaulter tracing, and linkages to health posts and health centers. Equipped with correct information, CVs are skilled to effectively communicate this to families.

“I usually create trust and form relationships with the households living in my kebele by speaking the truth and creating linkages with the health facility, helping needy families from the kebele contact the kebele administration and health workers. Since I am from the community, I do not have any problem to relate and interact with my community. As a woman Community Volunteer, I can use our women’s association to create the link smoothly.” (CV, Hayedimtu)

Stakeholders and partners identified the importance of strong supervision and training at all levels. This degree of supervision allowed CVs to perform their jobs effectively. Community volunteers self-reported strong supervision and good working relationships among themselves, their supervisors, and HDA volunteers. Community volunteers found their supervisors to be available to offer guidance and assistance.

“Yes, I frequently meet my supervisor and whenever I required support and guidance, I will go to my supervisor and take assistances. Without a doubt, I feel that I have adequate supervision.” (CV, Chereti)

All interviewed CVs reported receiving training on joining the program. This is called induction training. Initially, the CGPP trains the volunteers over 3 days on case definitions and on reporting and then reviews the roles of volunteers and facility-based workers in notification, investigation, and response. However, the amount of training after this orientation varied. Universally, CVs reported the desire for additional refresher training to strengthen their knowledge, including in other areas of health. These additional skills helped CVs to gain the trust of the community. They will also likely make it possible for CVs to fill other roles in their communities after the eradication of polio.

Community volunteers cited that building relationships based on trust, respect, and sound interpersonal communication is the primary reason for the hard-fought success.

“When we visit households, we greet them and give respect. We establish good rapport with them and teach about health packages. We take time to convince them on the next steps of health improvement; thus, respect, teaching, and convincing are our strategies.” (CV, Bench Maji)

“Personally, I did not receive any higher-level education, but I am sociable in my community. I have the ability to talk with other mothers, and I am committed to my community.” (CV, Aguna)

The communities select their CVs. This gives the CVs a network of connections and an intimate understanding of the needs, views, and understanding of community members that would not be possible for someone from the outside.

“The quality of CVs is that they are closely attached to their respective community … [and] CVs easily understand the outlook of the local community. These [traits] make them successful in their process of awareness creation.” (CV, Afder)

### Polio transition: future roles for the CGPP CVs.

As global eradication moves closer to reality, and as Ethiopia continues to look toward the future, stakeholders spoke of the importance of a planned and thoughtful transition. They were adamant that the structures and networks do not disappear once polio disappears.

“We have to gear our efforts toward enabling the government and the community to take over what we are doing and own the immunization program. And the legacy of polio must be utilized, it is very important. The coming five years are very decisive – the culmination point of all our work. Unless we are very good at handing over responsibility to the government, we are failing. This is the most challenging period. This is not easy. It is our burden and our task to show the paramount importance of taking over of all these functions by the government.” (Rotary International Consultant, Addis Ababa)

Not only are documenting and sharing the lessons learned in the polio eradication effort seen by those interviewed as important to the program’s legacy, but so is the preservation of the infrastructure—both physical and human. When asked about the future and the (presumed) forthcoming transition, participants saw routine immunization as a natural home for the resources and networks established by the CGPP. Working at the community level, as the CGPP has performed, will allow communities to have a voice in the transition. This will create buy-in and the most feasible solutions, according to participants.

“CGPP has provided for community engagement and community ownership, and for building working relationships between communities and health facilities. CGPP has made it possible for all partners to have a lower-level presence for all partners. This will help to sustain the activities in hard-to-reach areas. We must not lose this, and we must let others understand this.” (MOH, EPI team leader, Addis Ababa).

## DISCUSSION

Over the past 20 years, CVs have been at the heart of the success of the GPEI. They have worked tirelessly to promote vaccination, allay misconceptions, and change norms surrounding vaccination.^11,12,18,24^ In host countries, the CGPP has made a notable impact on the fight against polio, using local nongovernmental organizations, volunteers, and CVs to make inroads into communities that were previously forgotten or left unvaccinated. Polio and routine immunization rates have steadily increased in the CGPP implementation areas over the life of the CGPP in Ethiopia.

Household visits by community health workers are positively associated with the immunization status of children. A study in Ethiopia found that children were nearly twice as likely to be fully vaccinated if their household had been visited by a community health worker.^24^ Oral polio vaccine 3 coverage in the CGPP implementation areas in Ethiopia rose from 61.1% to 73.5% between 2012 and 2017, and the percentage of fully immunized children increased from 24.7% to 43.6%. These gains in coverage are particularly notable because they were achieved in some of the hardest-to-reach areas of Ethiopia.^21^ During the same 5-year period, the CGPP CVs contributed significantly to AFP surveillance by reporting 67% of all detected cases in the areas where they were working. Community volunteers also contributed to stronger AFP surveillance among remote, hard-to-reach, and migratory populations for whom health facility utilization is a challenge. During the same period, CVs identified and referred 306,432 women for tetanus immunization and 181,192 newborns for routine immunization, and they tracked 71,904 children younger than 1 year who had fallen behind on their immunization schedule.^21^

Through health education, mothers’ understanding of vaccination has increased, and this has helped mothers to make independent decisions. The routine immunization coverage in the CGPP implementation areas, as well as the utilization of antenatal care, has increased year after year. It is important to emphasize that CVs by themselves cannot vaccinate children (except for providing oral polio vaccination). Their role in increasing demand and providing referrals for vaccination can only make a notable difference if the communities have access to vaccines and to health service providers.

To support health systems’ strengthening, the CGPP continues to provide training for health personnel on topics such as the cold chain and vaccine timeliness. The CGPP supports vaccine outreach by providing fuel, funding personnel to travel to communities, and supporting mobile clinics for some hard-to-reach areas. However, additional health systems strengthening and facilitating access to health services and vaccination are needed to ensure that vaccine demand translates to vaccine provision.

Partnership, effective leadership, and grassroots interventions have been crucial to this success. The closely coordinated programming that the CGPP has achieved with the HDA volunteers and with the Ministry of Health has allowed the CGPP to support government systems instead of running ineffective parallel activities.^25^ Community volunteers work under the guidance and support of HEWs. This strategy reinforces regular supportive supervision, monthly review meetings, and written activity reports. This close integration also serves to motivate the CVs, who typically choose to remain in their roles for years at a time. Community volunteers have contributed to reductions in other diseases by promoting community participation and acceptance of positive health behaviors.

Community buy-in is central to the success of grassroots interventions for polio.^11^ The CGPP in Ethiopia has sought to establish community ownership by allowing communities to select their own volunteers. However, making inroads into communities and establishing trust was not an easy feat. It took concerted and persistent effort on the part of CVs. Strong interpersonal communication strategies can be effective not only in building community trust but also in the increasing vaccine uptake.^26^ Community volunteers in Ethiopia acquired strong interpersonal communication skills through training, experience, and learning from each other. This, along with behavior modeling and consistent long-term contact served to build trust and establish long-lasting relationships. Community volunteers serve without payment, which demonstrates their devotion and motivation and helps build trust and acceptance. It is this strong network of community relationships that can be used for future public health interventions in the hardest-to-reach areas of Ethiopia.

The contributions of the CGPP CVs have reached beyond polio immunization and surveillance. Community volunteers promote other important health-related behaviors such as referring pregnant women to the nearest health facility for antenatal care, tracking the status of routine immunizations in children, mobilizing communities for environmental health and personal hygiene activities, and identifying and referring cases of measles in the most remote areas. However, the full impact of CVs can only be realized in a functional health system that is able to fully support their efforts and respond to the referrals which they make.

## RECOMMENDATIONS

1. Community volunteers need ongoing support, especially for training and supervision. These are the underpinnings of success. Consistent contact with community members and providing them with correct, in-depth information are the building blocks of trust with the community. Failing to provide supervision and capacity building would damage the strong community network that has been established. Community volunteers, themselves, want and need further training and information outside of immunizations. Continued training will give them the ability to make stronger linkages and referrals to health facilities and services.
2. The CGPP should continue to work with partners and government entities to strengthen health systems, so that the increased demand for services they stimulate can be met. The CGPP should leverage its partnerships to lobby for and support health systems strengthening throughout Ethiopia, particularly in pastoralist areas that have traditionally been left behind. Strong linkages with the HDA and health facilities and with the government health system must continue and be strengthened.
3. The CGPP should continue to find innovative ways to engage and support mothers without adding significant burdens to their already heavy workload. One way to do this is by providing childcare during mothers’ meetings and holding meetings at times that do not conflict with the schedule of daily chores.
4. Engagement between CVs and men needs to continue and strengthen, particularly by involving male CVs. Male decision-making remains key to vaccination success (and is also important for child health success as well). The program should continue to focus on male engagement by ensuring that men have solid information about immunization and are actively involved in their children’s vaccination.
5. The CGPP needs to be thoughtful about transition. It needs to think carefully about how the CGPP experience can be applied in the Ethiopian context for the longer term beyond polio eradication. What are the key lessons for the Ethiopian context?
6. There is a need to document the formation of the CGPP’s partnerships and networks and their growth from the community to national levels.
7. Future CV involvement should extend beyond polio immunization and surveillance activities to reach a broader set of activities related to reducing the number of deaths from readily preventable or treatable conditions and diseases, particularly among mothers and children.
8. Continued health systems’ strengthening in Ethiopia should be a priority, including improving access to routine immunizations, polio campaigns, antenatal services, and other health services. Without improved access, the demand created for these services within communities will not translate to increased utilization, population coverage, and improved health.

## CONCLUSION

In Ethiopia, the CGPP CVs have been instrumental in the fight for polio eradication by overcoming many of the challenges and pressures faced by paid frontline health workers. By engaging local communities, sharing knowledge, acting as bridges to health posts, and pushing to reach the “unreachable,” CGPP CVs have established themselves as crucial assets in the community. Community members have come to rely on them as trusted sources of information. National and international stakeholders in polio eradication now recognize them as members of an extensive network that creates access to formerly inaccessible communities. Indeed, the success of CVs is rooted in their commitment to their communities and in the high value they give to maintaining these trusted relationships. The CGPP has remained committed to fostering partnerships and integrating the CVs with existing government health worker cadres. However, in conjunction with this commitment, it is vital that CVs continue to receive adequate training and supervision and that the government continues to strengthen health systems to support the needs of communities. Moreover, once the global eradication of polio becomes a reality, leveraging the future roles of CGPP CVs toward other health priorities, including strengthening routine immunization, will become a moral imperative.

## Footnotes

† Pastoralist—a nomadic way of life based on the raising and herding of livestock.

## References

[1] WHO, 2019 Poliomyelitis Fact Sheet. Available at: http://www.who.int/mediacentre/factsheets. Accessed May 14, 2019.

[2] Centers for Disease Control and Prevention (CDC), 2000 Progress toward poliomyelitis eradication–Ethiopia, 1997–August 2000. MMWR Morb Mortal Wkly Rep 49: 867–870. 11032094

[3] Abraham K Bisrat F Fantahun M Asres M Kidane L Rogie B, 2013 Acute flaccid paralysis surveillance status and community awareness in pastoralist and semi-pastoralist communities of Ethiopia. Ethiop Med J 51 (Suppl 1): 13–20. 24380203

[4] WHO, 2015 Polio Eradication in Ethiopia: Progress in 2014. Addis Ababa, Ethiopia: World Health Organization.

[5] Mesfin G Schluter W Gebremariam A Benti D Bedada T Beyene B Yigzaw A Taddess Z Mbakuliyemo N Babaniyi O, 2008 Polio outbreak response in Ethiopia. East Afr Med J 85: 222–231. 18814532 10.4314/eamj.v85i5.9616

[6] Tegegne AA Braka F Shebeshi ME Aregay AK Beyene B Mersha AM Ademe M Muhyadin A Jima D Wyessa AB, 2018 Characteristics of wild polio virus outbreak investigation and response in Ethiopia in 2013–2014: implications for prevention of outbreaks due to importations. BMC Infect Dis 18: 9. 29304745 10.1186/s12879-017-2904-9 PMC5756383

[7] World Bank, 2018 Urban Population. Available at: https://data.worldbank.org/indicator/SP.URB.TOTL.IN.ZS. Accessed May 14, 2019.

[8] GPEI, 2018 Wild Poliovirus List. Available at: http://polioeradication.org/polio-today/polio-now/wild-poliovirus-list/. Accessed May 14, 2019.

[9] Asegedew B Tessema F Perry H Bisrat F, 2019 The CORE Group Polio Project’s community volunteers and polio eradication in Ethiopia: self-reports of their activities, knowledge, and contributions. Am J Trop Med Hyg 101 (Suppl 4): 45–51. 31760977 10.4269/ajtmh.18-1000 PMC6776091

[10] CGPP, 2016 Enhancing AFP Surveillance in Ethiopia Somali Region: Some Good Practices in the Implementation of BMGF Supported Project. Ethiopia: CORE Group Polio Project. Available at: https://coregroup.org/wp-content/uploads/2017/08/CGPP_Ethiopia_Good_practice.pdf. Accessed May 14, 2019.

[11] Nasiru SG Aliyu GG Gasasira A Aliyu MH Zubair M Mandawari SU Waziri H Nasidi A El-Kamary SS, 2012 Breaking community barriers to polio vaccination in northern Nigeria: the impact of a grass roots mobilization campaign (Majigi). Pathog Glob Health 106: 166–171. 23265374 10.1179/2047773212Y.0000000018 PMC4001576

[12] Deutsch N Singh P Singh V Curtis R Siddique AR, 2017 Legacy of polio-use of India’s social mobilization network for strengthening of the universal immunization program in India. J Infect Dis 216 (Suppl 1): S260–S266. 28838190 10.1093/infdis/jix068 PMC5854010

[13] Solomon R, 2019 Involvement of civil society in India’s polio eradication: lessons learned. Am J Trop Med Hyg 101 (Suppl 4): 15–20. 10.4269/ajtmh.18-0931 PMC6776100 31760980

[14] Kamadjeu R 2015 Immunizing nomadic children and livestock–experience in north east zone of Somalia. Hum Vaccin Immunother 11: 2637–2639. 26365693 10.1080/21645515.2015.1038682 PMC4685689

[15] Kidanne L Bisrat F Dinku B Lynch M Fantahun M, 2013 Newborn tracking for polio birth dose vaccination in pastoralist and semi-pastoralist CORE Group Polio Project implementation districts (woredas) in Ethiopia. Ethiop Med J 51 (Suppl 1): 1–12. 24380202

[16] Curry DW Bisrat F Coates E Altman P, 2013 Reaching beyond the health post: community-based surveillance for polio eradication. Dev Pract 23: 69–78.

[17] Curry DW Perry HB Tirmizi SN Goldstein AL Lynch MC, 2014 Assessing the effectiveness of house-to-house visits on routine oral polio immunization completion and tracking of defaulters. J Health Popul Nutr 32: 356–366. 25076672 PMC4216971

[18] Kowli SS Bhalerao VR Jagtap AS Shrivastav R, 1990 Community participation boosts immunization coverage. World Health Forum 11: 169–172. 2271093

[19] Caglia J Kearns A Langer A, 2014 Health Extension Workers in Ethiopia: Delivering Community-Based Antenatal and Postnatal Care. Available at: https://cdn2.sph.harvard.edu/wp-content/uploads/sites/32/2014/09/HSPH-Ethiopia4.pdf. Accessed May 14, 2019.

[20] Goldstein A Kidanne L Bisrat F, 2018 Integrating Community Volunteers and Health Extension Workers to Increase Scalability and Sustainability of the CORE Group Polio Project (CGPP) in Ethiopia. Poster Presentation. Available at: https://coregroup.org/wp-content/uploads/2017/08/Increase_Scalability_and_Sustainability_Poster.pdf. Accessed May 14, 2019.

[21] Stamidis K Bologna L Losey L, 2018 CORE Group Polio Project (CGPP) Final Evaluation Report 2017. Available at: https://coregroup.org/wp-content/uploads/2018/06/CGPP-Evaluation-Report-FINAL-5-10-2018.pdf. Accessed May 14, 2019.

[22] Tessema F Bisrat F Kidane L Assres M Tadsse T Asegedew B, 2019 Improvements in polio vaccination status and knowledge about polio vaccination in the CORE Group Polio Project implementation areas in pastoralist and semi-pastoralist regions in Ethiopia. Am J Trop Med Hyg 101 (Suppl 4): 52–58. 10.4269/ajtmh.19-0022 PMC6776097 31760976

[23] Ulin P Robinson E Tolley E, 2005 Qualitative Methods in Public Health: A Field Guide for Applied Research. San Francisco, CA: Jossey-Bass.

[24] Mohamud AN Feleke A Worku W Kifle M Sharma HR, 2014 Immunization coverage of 12–23 months old children and associated factors in Jigjiga district, Somali National Regional State, Ethiopia. BMC Public Health 14: 865. 25146502 10.1186/1471-2458-14-865 PMC4158082

[25] Dinku B Kumie A Bisrat F, 2013 Linking community volunteer surveillance focal persons with health extension workers on polio surveillance. Ethiop Med J 51 (Suppl 1): 71–76. 24380209

[26] Ozohu-Suleiman Y, 2010 Media and interpersonal persuasions in the polio eradication campaign in northern Nigeria. J Public Health Afr 1: e2. 28299036 10.4081/jphia.2010.e2 PMC5345393

